# Murine Salivary Amylase Protects Against *Streptococcus mutans*-Induced Caries

**DOI:** 10.3389/fphys.2021.699104

**Published:** 2021-07-02

**Authors:** David J. Culp, Bently Robinson, Melanie N. Cash

**Affiliations:** Department of Oral Biology, College of Dentistry, University of Florida, Gainesville, FL, United States

**Keywords:** microbiome, *Streptococcus*, aggregation, animal model, infectious disease, innate immunity

## Abstract

Saliva protects dental surfaces against cavities (i. e., dental caries), a highly prevalent infectious disease frequently associated with acidogenic *Streptococcus mutans*. Substantial *in vitro* evidence supports amylase, a major constituent of saliva, as either protective against caries or supporting caries. We therefore produced mice with targeted deletion of salivary amylase (Amy1) and determined the impact on caries in mice challenged with *S. mutans* and fed a diet rich in sucrose to promote caries. Total smooth surface and sulcal caries were 2.35-fold and 1.79-fold greater in knockout mice, respectively, plus caries severities were twofold or greater on sulcal and smooth surfaces. In *in vitro* experiments with samples of whole stimulated saliva, amylase expression did not affect the adherence of *S. mutans* to saliva-coated hydroxyapatite and slightly increased its aggregation in solution (i.e., oral clearance). Conversely, *S. mutans* in biofilms formed in saliva with 1% glucose displayed no differences when cultured on polystyrene, but on hydroxyapatite was 40% less with amylase expression, suggesting that recognition by *S. mutans* of amylase bound to hydroxyapatite suppresses growth. However, this effect was overshadowed *in vivo*, as the recoveries of *S. mutans* from dental plaque were similar between both groups of mice, suggesting that amylase expression helps decrease plaque acids from *S. mutans* that dissolve dental enamel. With amylase deletion, commensal streptococcal species increased from ~75 to 90% of the total oral microbiota, suggesting that amylase may promote higher plaque pH by supporting colonization by base-producing oral commensals. Importantly, collective results indicate that amylase may serve as a biomarker of caries risk.

## Introduction

Dental caries is among the most prevalent infectious diseases in developed as well as developing nations (Satcher, 2000; Marcenes et al., 2013), particularly among underprivileged groups (Bowen, 2002). Caries results from the demineralization of the tooth enamel by organic acids produced by acidogenic oral pathogens such as *Streptococcus mutans* during the fermentation of carbohydrates (Bowen et al., 2018). With increasing frequency of exposure to sucrose (i.e., table sugar) or high-fructose corn syrup used in many soft drinks and processed foods, *S. mutans* within dental biofilms produces organic acids, resulting in the prolonged acidification and progressive demineralization of enamel surfaces. *S. mutans* can also metabolize sucrose to extracellular polysaccharides by three secreted glucosyltransferases (i.e., GtfB, GtfC, and GtfD) and by secreted fructosyltransferase. GtfB and GtfC are important virulence factors of *S. mutans* (Kuramitsu, 1993) as they are responsible for the production of insoluble glucans produced by cell-associated enzymes in addition to secreted enzymes. Insoluble glucans establish an extracellular polysaccharide matrix that contributes to the structural integrity of cariogenic dental biofilms and further promotes the adhesion and accumulation of mutans streptococci *via* cell-associated glucan-binding proteins (Vacca Smith et al., 2007; Hannig et al., 2008; Bowen and Koo, 2011). GtfD produces soluble glucans shown to play a role in the formation of caries on the smooth surfaces of teeth (Yamashita et al., 1993). Fructosyltransferase catalyzes the synthesis of high-molecular-mass fructans from sucrose, which, along with glucans, can serve as an energy source when dietary carbohydrates are not available (Vacca Smith et al., 2007; Hannig et al., 2008; Bowen and Koo, 2011). Caries is further characterized as a complex polymicrobial biofilm disease associated with the competitive interplay between matrix-forming and acidogenic opportunistic pathogens such as *S. mutans* and oral commensals (Bowen et al., 2018). The decrease in pH within dental biofilms results in a microbial community shift that favors colonization by more acid-tolerant species such as *S. mutans* at the expense of less acid-tolerant non-pathogenic commensals (Bowen et al., 2018). *S. mutans* is not only highly associated with dental caries but is also a causative agent of bacterial endocarditis and is found in atheromatous plaques (Kleinstein et al., 2020), indicating an interconnection between dental health and cardiovascular diseases.

Saliva plays a major role in the innate immunity of the oral cavity, protecting teeth through its flushing action, buffering of acids, promotion of remineralization, bacteriostatic and bactericidal activities, and functioning in selective adherence of commensals and in the clearance of acidogenic microorganisms (Scannapieco, 1994; Van Nieuw Amerongen et al., 2004; Tabak, 2006; Bowen et al., 2018). Three pairs of “major” salivary glands (parotid, submandibular, and sublingual) are linked to the oral cavity through relatively long excretory ducts, whereas just under the oral epithelium are numerous smaller “minor” glands, named according to their anatomical locations (i.e., labial, lingual, palatal, buccal, and minor sublingual) (Pinkstaff, 1980). The production of saliva involves the coordinated neural activation of these diverse glandular structures, each contributing a different subset of exocrine constituents (Pinkstaff, 1980). One of the most abundant constituents of saliva is α-amylase that, in humans, is found in serous cells of the parotid, submandibular, and minor glands, such as von Ebner's glands (Pinkstaff, 1980). The salivary concentration of amylase increases with mastication during a meal (Rohleder et al., 2006). The enzyme catalyzes the hydrolysis of α-1,4-glucosidic bonds of polysaccharides such as starch and glycogen to yield maltodextrin, maltose, and glucose (Koyama et al., 2000). Post-translational modifications of amylase include *N*-glycosylation, addition of trace levels of carbohydrate, or deamination of glutamine and asparagine to glutamate and aspartate, respectively, thus contributing to multiple isozymes in saliva (Zakowski and Bruns, 1985).

There is evidence to suggest that salivary amylase functions in the protection against caries. Salivary amylase plays a role in the formation of dental biofilms. *In vitro*, α-amylase binds to hydroxyapatite *via* several acidic residues within its N-terminal domain (Johnsson et al., 1993; Ramasubbu et al., 1996). Hydroxyapatite, crystals of calcium phosphate salts, accounts for about 95% of the tooth enamel and is therefore used *in vitro* as a surrogate for tooth enamel (Siqueira et al., 2007). Amylase is localized within the acquired enamel pellicle (AEP) that results from the rapid interactions between a clean enamel surface with saliva (Rolla and Rykke, 1994; Lendenmann et al., 2000; Deimling et al., 2004). The AEP helps to regulate enamel mineralization and lubrication and influences the initial colonization by select oral commensals (Siqueira et al., 2012). Amylase is also a constituent of mature biofilms on teeth (i.e., dental plaque) (Lendenmann et al., 2000), and many species of commensal oral streptococci bind amylase *via* an amylase-binding protein (ABP) (Nikitkova et al., 2013). When within plaque or the AEP, α-amylase bound to streptococci expressing ABP remains enzymatically active, allowing commensal streptococci expressing ABP to have preferred access to the enzymatic products of bound amylase for catabolism, thereby enhancing their competitiveness and/or providing metabolic substrates for nearby non-ABP-expressing bacteria (Douglas et al., 1992; Scannapieco et al., 1995; Ramasubbu et al., 1996; Vacca Smith and Bowen, 2000; Aizawa et al., 2009; Nikitkova et al., 2013). Because many streptococci expressing ABP are pioneer colonizers of the AEP, amylase is therefore thought to function in the establishment of dental biofilms and in contributing to the maintenance of a healthy dental plaque (Nikitkova et al., 2013). Amylase may also promote interbacterial interactions within dental biofilms through dimerized amylase (Fisher et al., 2006) or through different species each using an ABP with a distinctive amylase-binding domain (Nikitkova et al., 2013). Amylase also has non-metabolic effects. For example, the interaction of amylase with the pioneer colonizer *Streptococcus gordonii* results in differential gene expression and promotes increased growth and resistance to low pH (Nikitkova et al., 2012).

Bacterial agglutination (or aggregation) in saliva and subsequent clearance as saliva is swallowed is an important mechanism to help control resident oral bacteria. Interestingly, subjects whose whole resting saliva displayed both high agglutination and high adherence to hydroxyapatite by the oral commensal *Streptococcus crista*, the dental pathogen *S. mutans*, and the periodontal pathogen *Aggregatibacter actinomycetemcomitans* were correlated with lower caries scores compared to those with lower agglutination and adherence (Rudney and Staikov, 2002), suggesting that the efficient agglutination and promotion of dental adherence are both required in controlling the oral microbiota for effective protection against caries. Oral commensals expressing ABP may therefore be cleared *via* direct interaction with salivary amylase (Nikitkova et al., 2013), whereas other oral species may be cleared indirectly through the complexation of amylase with many different proteins in saliva, including MUC5B, MUC7, salivary agglutinin, proline-rich proteins, histatin, and statherin (Soares et al., 2004; Crosara et al., 2018). In particular, both MUC7 and salivary agglutinin, when in solution, were shown to bind to *S. mutans* (Liu et al., 1999; Tabak, 2006; Ligtenberg et al., 2007) and therefore may agglutinate and clear this oral pathogen as part of amylase-containing heterotypic complexes, especially since amylase alone in solution was unable to bind *S. mutans* (Nikitkova et al., 2013). Amylase is also a component of salivary micelles, large 300- to 400-nm diameter globular macromolecular structures suspended in saliva that, in addition to amylase, were shown to contain MUC7, secretory IgA, lactoferrin, glycosylated proline-rich proteins, and lysozyme and therefore may also promote the clearance of *S. mutans* (Rykke et al., 1997; Soares et al., 2004). Moreover, a complex of amylase and an unidentified high-molecular-weight glycoprotein in human saliva was shown to bind selectively to cell-associated glucosyltransferases (Gtfs) of *S. mutans via* amylase and to inhibit Gtf activity (Jespersgaard et al., 2002).

Alternatively, there is evidence supporting a role for salivary amylase in the promotion of caries. In animal studies with *S. mutans*, a high-starch diet in the absence of sucrose elicited very low levels of caries (Michalek et al., 1977). Additionally, when cultured with starch in the absence of sucrose, *S. mutans* was shown to produce low amounts of biofilms and insoluble glucans on hydroxyapatite discs coated with saliva (Duarte et al., 2008). Possibly contributing to the low cariogenicity of starch is the observation that surface-absorbed Gtf enzymes released by *S. mutans* markedly inhibited amylase activity (Vacca-Smith et al., 1996). However, despite being unable to directly catabolize starch, *S. mutans* was shown to utilize maltose and maltodextrins for the production of acid, plus glucose for the synthesis of insoluble extracellular glucans by GtfB, while maltose and maltodextrins serve as acceptors during glucan synthesis by Gtf enzymes (Vacca-Smithet al., 1996; Klein et al., 2010; Sato et al., 2013). Furthermore, a high-starch diet required as little as 0.1% sucrose to elicit significant caries in gnotobiotic rats (Michalek et al., 1977). *In vitro*, starch, in combination with sucrose, markedly enhanced the cariogenic potential of *S. mutans* biofilms compared to sucrose alone, as evidenced by the higher biofilm acidogenicity, greater enamel demineralization, upregulation of genes associated with sugar uptake and metabolism, increased *S. mutans gtfB* messenger RNA (mRNA) and protein, and a higher proportion of insoluble glucans with distinctive branched glucose structures that were more resistant to breakdown by glucan endo-1,3-alpha-glucosidase and promote greater adherence of *S. mutans* (Vacca-Smith et al., 1996; Duarte et al., 2008; Klein et al., 2009, 2010; Botelho et al., 2016). Moreover, amylase, in the presence of sucrose, was shown to enhance both the transferase and sucrase activities of extracellular *S. mutans* GtfB in culture supernatants (Chaudhuri et al., 2007). Dietary starch tends to accumulate and be retentive on teeth; thus, in the presence of underlying *S. mutans* and amylase, retained starch may promote the initiation and progression of carious lesions (Lingstrom et al., 2000). Interestingly, a clinical isolate of *S. mutans* from a caries-active child was shown to express a 65-kDa fimbrial protein that selectively bound to amylase in saliva (Ray et al., 1999), suggesting that this protein may promote dental colonization by *S. mutans*. Combined evidence therefore supports salivary amylase acting in a cooperative manner with sucrose and starch to promote colonization by *S. mutans*, glucan synthesis, biofilm acidogenicity, and fostering caries initiation and severity (Bowen and Koo, 2011).

Collective evidence therefore suggests that amylase may function through multiple mechanisms to either promote or attenuate the incidence and progression of caries mediated by *S. mutans*. However, the net effects of the loss of salivary amylase on the innate protective functions of saliva *in vivo* have not been explored. Because mice and humans share many of the core proteins in saliva, including salivary α-amylase (Karn et al., 2013), the impact of targeted deletion of salivary amylase on the development of caries upon challenge of mice with *S. mutans* and a highly cariogenic diet was determined. In addition, saliva from knockout mice was compared to that of wild-type mice, *in vitro*, for interactions with *S. mutans* in assays of innate immune-protective mechanisms. Further experiments evaluated whether the deletion of salivary amylase expression altered the composition of the oral microbiota. The results clearly demonstrated that salivary amylase is markedly protective against dental caries induced by *S. mutans*; these are discussed with respect to putative protective mechanisms based on the outcomes of *in vitro* assays and changes in the oral commensal microbiota.

## Materials and Methods

### Materials

Unless indicated, all materials were from Invitrogen, Carlsbad, CA, USA. All kits were used according to the manufacturer's instructions.

### Generation of *Amy1* Knockout Mice

The University of Florida IACUC committee approved all animal procedures (IACUC Study #201202499), following the National Institutes of Health Guide for the Care and Use of Laboratory Animals. Generation and screening of embryonic stem (ES) cell clones and the production of chimeric mice were conducted at the University of Cincinnati Gene Targeted Mouse Service. Incorporation of the targeting vector by recombination results in excision of the first two coding exons and part of the third coding exon of *Amy1*, disrupting the expression of salivary and hepatic α-amylase enzymes (see **Figure 2**). The vector was cloned into Electro 10 Blue cells (Agilent Technologies, La Jolla, CA, USA), sequenced, and the purified plasmid DNA linearized with *Asc*I. The linearized plasmid was electroporated into 129svev (CMT1-1) ES cells and the clones (after G418 selection) were screened for correct vector integration by Southern blot analysis. Targeted 129svev ES cell clones were injected into blastocysts (C57BL/6 × DBA). Three male chimeras (129S/C57B6J) from a single ES cell clone were mated to Black Swiss females (NIHBS) and produced 20 heterozygous F1 agouti mice (10 males and 10 females) that were transferred to the University of Florida. Two heterozygous F1 agouti females were mated to a FLPeR male (129S4/SvJaeSor-Gt(ROSA)26Sor^tm1(FLP1)Dym/J^) from Jackson Labs to delete the *neo* cassette. *Amy1*^+/−^ (*neo*^−^) males were used to initiate backcrosses to NFS/N mice with selected deletion of the R26^Fki^ allele. N5 heterozygotes were intercrossed to establish two closed colonies, one *Amy1*^−/−^ and the other *Amy1*^+/+^ controls. Pups were genotyped using tail genomic DNA (Wizard Genomic DNA purification kit; Promega, Madison, WI, USA). The PCR primers and conditions are given in Supplementary Table 1. The strain NFS/N.Cg-*Amy1*^*tm*1.1*Culp*^/Mmucd (#36650-UCD) is available for distribution from the Mutant Mouse Resource & Research Center (https://www.mmrrc.org). Mouse colonies were raised under ABSL2 conditions by vivarium staff in a closed no-investigator access facility, and we conducted experiments under ABSL2 conditions, all specifically designed to help prevent additional bacteria from colonizing the oral cavity and influencing the results. Mice were euthanized by CO_2_ inhalation followed by cervical dislocation. Unless indicated, the tissues were excised, blotted on filter paper, flash frozen, and stored in liquid nitrogen.

### Anatomical Comparisons Between Mice

Five mice of each sex and genotype were examined at 7 weeks of age for outward body characteristics (body shape and size; the number of digits and claws on each limb; fur color and texture on the back and belly; skin appearance; size and shape of the head; the position, size, and shape of ears; the size, position, clarity, and color of the eyes; the number, position, size, and color of the teeth; whiskers; the size and shape of limbs, genitals, anus, and mammary glands; the color and hardness of feces; and the coloration of urine stains of the litter) and for any obvious differences in internal organs (palate, tongue, buccal mucosa, major salivary glands, esophagus, stomach, duodenum, pancreas, spleen, ileum, jejunum, cecum, colon, liver, gallbladder, kidney, adrenal glands, urinary bladder, tracheolarynx, thymus, lungs, heart, lymph nodes, mesentery, scrotum, testes, epididymis, seminal vesicle, bulbourethral gland, penis, ovaries, uterus, vagina, Harderian glands, lacrimal glands, and brain).

### RT-PCR

Procedures were as described previously (Das et al., 2009). Briefly, RNA was isolated from frozen tissues and DNase I-treated RNA (5 μg) was reverse transcribed with random primers. The PCR conditions and specific primers used are in Supplementary Table 1.

### SDS-Page

Frozen tissues (10–20 mg wet weight) were placed in 300–500 μl of sample buffer and then sonicated (4 × 10-s pulses at 30-s intervals) with a Branson Digital Sonifier 250 at 30% amplitude (Branson Ultrasonic Corp., Danbury, CT, USA), boiled for 10 min, and centrifuged (10,000 × g, 10 min) at 4°C. Saliva or supernatant volumes equivalent to the indicated wet weight of the original tissue were applied directly to NuPAGE 4–12% Bis-Tris, NuPAGE 3–8% Tris-acetate, or Novex 10–20% Tricine gels. Gels were stained for proteins with Coomassie blue using standard protocols, or to assay highly glycosylated glycoproteins, the gels were stained with Alcian blue with subsequent silver enhancement (Jay et al., 1990). Digital images of the gels were obtained with a Scion Grayscale 1394 Digital Camera (Fotodyne, Hartland, WI, USA).

### Westerns Blots

Saliva (15 μl) or tissue samples (200 μg wet weight) from wild-type (WT) and knockout (KO) mice were run on 4–12% SDS-PAGE gels, blotted into PVDF membranes, and probed with rabbit polyclonal anti-α-amylase (1:2,500 dilution; A8373, Sigma-Aldrich, St. Louis, MO, USA). Bound antibody was detected using WesternBreeze Chemiluminescent Western Blot Immunodetection Kit and BioMax Light film (Eastman Kodak Co., Rochester, NY, USA).

### Collection of Stimulated Whole Saliva From Mice

Saliva collection was as described previously (Culp et al., 2011). Briefly, mice 5–6 weeks of age (three males and two females of each genotype) were anesthetized (75 mg/kg ketamine plus 8 mg/kg xylazine, i.p.), the saliva flow stimulated (subcutaneous 5 mg/kg pilocarpine/0.5 mg/kg isoproterenol), and saliva collected from the cheek pouch at 5-min intervals over a 20-min period. To collect stimulated saliva for biochemical analyses and *in vitro* assays, male and female mice between 6 and 10 weeks of age were anesthetized and stimulated as described above and saliva collected on ice over 20 min, resulting in about 18–22 μl total saliva that was immediately frozen at −80°C. For each biochemical analysis or *in vitro* assay, 15 μl of saliva from each of an equivalent number of samples from male and female mice was quickly thawed, pooled, and used immediately.

### Assay of the Effects of Amylase on *S. mutans* Within Biofilms

Assays were performed as described previously (Culp et al., 2015). For this and all *in vitro* assays with whole stimulated saliva, we used *S. mutans* UA159 containing the ΩKm element integrated into the *gtfA* gene with the vector pBGK (Wen and Burne, 2001), which does not alter carbohydrate metabolism, except for melibiose and raffinose (Barletta and Curtiss, 1989), nor is caries development by this strain affected (Tanzer et al., 2006). The use of this strain and kanamycin in *in vitro* assays allowed avoiding centrifugation or filtration of whole stimulated saliva to remove inherent microorganisms and to clarify saliva. Centrifugation or filtration removes most of the gel phase of saliva that contains gel-forming high-molecular-weight mucin glycoproteins in addition to associated salivary proteins and microorganisms (Culp, unpublished observations). The collected saliva produced no colonies under aerobic or anaerobic conditions on blood agar containing 1 mg/ml kanamycin (data not shown). Briefly, the assays were carried out in 96-well polystyrene plates with or without insertion of hydroxyapatite discs (Clarkson Chromatography Products Inc., South Williamsport, PA, USA). Samples of mouse saliva were resuspended to a concentration of 63% (*v*/*v*) in saliva buffer (SB; 50 mM KCl, 1.0 mM KPO_4_, 1.0 mM CaCl_2_, and 0.1 mM MgCl_2_, pH 6.5) plus 1 mg/ml kanamycin (SBK). Wells received 100 μl 63% saliva and the plates incubated for 30 min at 37°C. Mid-log phase cultures of kanamycin-resistant *S. mutans* UA159 were diluted 1:100 in pre-warmed semi-defined biofilm medium (BM; basic salts, vitamins, key amino acids, and casamino acids) (Lemos et al., 2010) containing 1% glucose plus 1 mg/ml kanamycin and incubated for 4 h at 37°C in air−5% CO_2_. Approximately 10^6^ cells in 150 μl were then added to the wells to give a final saliva concentration of 25%. After 24 h at 37°C in air−5% CO_2_, the medium was decanted and the biofilms washed twice gently with 200 μl sterile 120 mM NaCl to remove planktonic and loosely bound cells. The biofilms were stained with 200 μl of 5 μM of the cell-permeant nucleic acid stain SYTO 13 in the dark for 25 min at room temperature and the fluorescence determined. All experimental conditions were performed in triplicate.

### Assay of the Effects of Amylase on the Aggregation of *S. mutans*

Assays were as described previously (Culp et al., 2015). Briefly, cell suspensions of kanamycin-resistant *S. mutans* UA159 were mixed with whole stimulated saliva diluted in SBK to give final concentrations as indicated (*v*/*v*). Initial cell suspensions (OD_600_ ≈ 0.65) were transferred to a spectrophotometer with a temperature-controlled multicuvette positioner set at 37°C. After 5 min, the OD_600_ of each sample was recorded at 10-min intervals over a 2-h period. Percent aggregation (percent decrease in OD_600_) was calculated as (OD_600_ at 0 min – OD_600_ at 120 min)/(OD_600_ at 0 min) × 100.

### Assay of the Effects of Amylase on the Adherence of *S. mutans*

Adherence assays were as described previously (Culp et al., 2011) using hydroxyapatite discs in 96-well plates and kanamycin-resistant *S. mutans* UA159. Wells received 100 μl diluted whole stimulated saliva or buffer (background controls) and the plates incubated for 1 h at 37°C with rotary shaking and washed twice with SBK. *S. mutans* in SBK (10^8^ cells in 100 μl) were added to the wells and incubated for 1 h at 37°C, then washed with SB. Adherent cells were quantified using SYTO 13 and a standard curve with a linear range of 10^8^-10^4^ cells. Values were normalized to the mean for cells bound to discs without saliva (100%). All experimental conditions were performed in triplicate.

### Caries Experimental Protocol and Scoring

The caries protocol was similar to that reported previously (Culp et al., 2015). Briefly, timed pregnancies were established using 20 breeding pairs each of homozygous NFS/NCr and *Amy1*^−/−^ mice and the pups inoculated at 16–17 days of age with ~10 μl (10^8^ CFU) of *S. mutans* UA159. The diet was converted to powdered Diet 2000 (56% sucrose, 28% skimmed milk powder, 6% whole wheat flour, 4% brewer's yeast, 3% alfalfa meal, 2% NaCl, and 1% desiccated liver; Harlan-Teklad, Madison, WI, USA) with 5% sucrose water. At 21 days of age, pups were weaned and caged in pairs (10 pairs per group) with non-littermates of the same sex. Mice were weighed weekly and sacrificed 7 weeks after the initial inoculation. The left and right mandibles were removed aseptically and assayed for bacterial colonization of the molars. The buccal, lingual, and proximal surfaces of all mandibular and maxillary molars were scored for smooth surface and sulcal caries using Larson's modification of the Keyes' scoring system (Keyes, 1958; Larson, 1981). The scores were compared by ANOVA with the Tukey–Kramer *post*-*hoc* test. To stabilize variances, caries scores were first expressed as a percentage of their maximum possible values (124 for total smooth surface caries, 54 for total buccal or lingual caries, 16 for proximal caries, and 48 for total sulcal caries) and then transformed to their arcsine square root.

### Recoveries of *S. mutans* and Total Bacteria From Molar Biofilms

The mandibles from 10 mice in each caries group were aseptically extracted and underwent dissection to remove loose tissue on the bone near the molars. The bone was sectioned about 2 mm anterior to the first molar and 2 mm posterior to the third molar. Each pair of prepared mandible/molars from each mouse were sonicated on ice in 5 ml of ice-cold sterile 0.9% saline using three pulses of 10 s with 30-s intervals at 50% power with a Branson Digital Sonifier 250. Sonicates from two non-cagemates were pooled. From each 10 ml, 200 μl was used to prepare 10-fold serial dilutions that were plated (50 μl per 60-mm plate) onto mitis salivarius–bacitracin (MSB) agar (Emilson and Bratthall, 1976) and onto blood agar and cultured aerobically to estimate colonization of the molars by *S. mutans* (MSB agar) and by total bacteria (blood agar). The colony forming units (CFUs) were then determined.

### Comparison of Indigenous Oral Bacteria

Procedures were as described previously (Culp et al., 2015). Oral swabs from 10 mice of each genotype (8–10 weeks of age) were streaked on sheep blood agar plates and incubated under an atmosphere of 95% air−5% CO_2_ and the total recovered CFUs for each colony morphotype determined. Subsequent colony isolates from each of three mice of each genotype were subjected to colony PCR using degenerate primers (Paster et al., 2001) complementary to the 5′- and 3′-ends of the *16S rRNA* gene. Consensus sequences were compared to reference sequences in the Human Oral Microbiome Database (HOMD 16S rRNA RefSeq 15.1) (Chen et al., 2010) and the Ribosome Database Project, release 11.5 (Cole et al., 2014). Alignments were performed *via* the BLAST server at HOMD (www.homd.org/index.php).

### Statistics

Comparisons were conducted as described using Prism 8.0 software (GraphPad Software, Inc., San Diego, CA, USA).

## Results

### Amylase Genes in Humans and Mice

**Figure 1:**
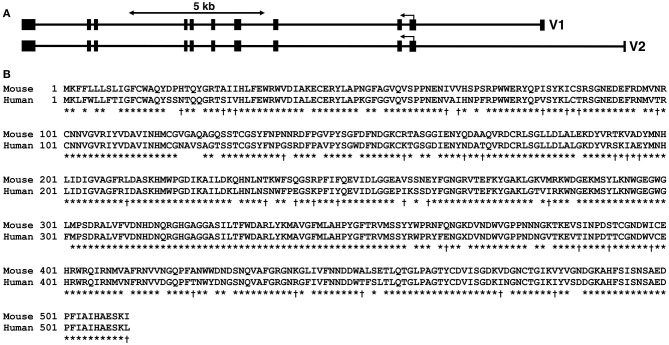
*Amy1* genomic organization in mice and comparison of murine with human salivary α-amylase protein sequences. **(A)** Genomic organization of the murine *Amy1* gene along the negative strand of mouse chromosome 3. The two transcripts, variant 1 (*V1*, NM_007446.2) and variant 2 (*V2*, NM_001110505.1), are each encoded by unique first untranslated exons, but share the same ten 3′-exons. The translation start codon is within exon 2, which encodes the first 56 residues including the signal peptide of 15 residues. Both transcripts thus encode the same protein. **(B)** ClustalW alignment of mouse Amy1 (NP_001110505.1) and human AMY1 (NP_004029.1) protein sequences. The gene products share 83.8% identity (*asterisk*) and an additional 6.4% similarity (*dagger*). The predicted signal peptide is indicated by the *line* (SignalP v5.0; Almagro Armenteros et al., 2019). The two genes are syntenic (*3F3*, mouse; *1p21*, human). ClustalW pairwise parameters: open gap penalty, 10; extend gap penalty, 0.1; delay divergent, 40%; gap distance, 8; similarity matrix, blosum.

In humans, α-amylases are encoded by separate genes clustered on human chromosome 1 p21 and include *AMY1* (i.e., salivary α-amylase), *AMY2A* (i.e., pancreatic α-amylase), and *AMY2B* expressed in liver and blood leukocytes (Tricoli and Shows, 1984; Koyama et al., 2001; Hokari et al., 2002). The murine *Amy1* gene specifies two mRNAs distinguished by different starting and non-coding exons located upstream of the initial coding exon (Young et al., 1981). Both gene products therefore encode the same protein, but are expressed separately in the salivary glands (isoform V2) and liver (isoform V1) (see Figure 1A). However, the liver promoter is weak compared to the salivary promoter (Schibler et al., 1982). The murine *Amy2* gene is represented by six genes immediately downstream of *Amy1*, each encoding mRNA isoforms expressed in the pancreas (Hagenbuchle et al., 1980). However, very low levels of *Amy2* message have been detected in the intestine, stomach, spleen, and liver (MacKenzie and Messer, 1976; Skude and Mardh, 1976; Samuelson et al., 1988). Four isoforms of Amy2 are predicted to encode identical full-length proteins that are highly homologous to Amy1 (see Supplementary Figure 1). More importantly, Amy1-predicted proteins in mice and humans display 83.8% identity and an additional 6.4% similarity (Figure 1B).

### Production and Characterization of *Amy1* Knockout Mice

**Figure 2:**
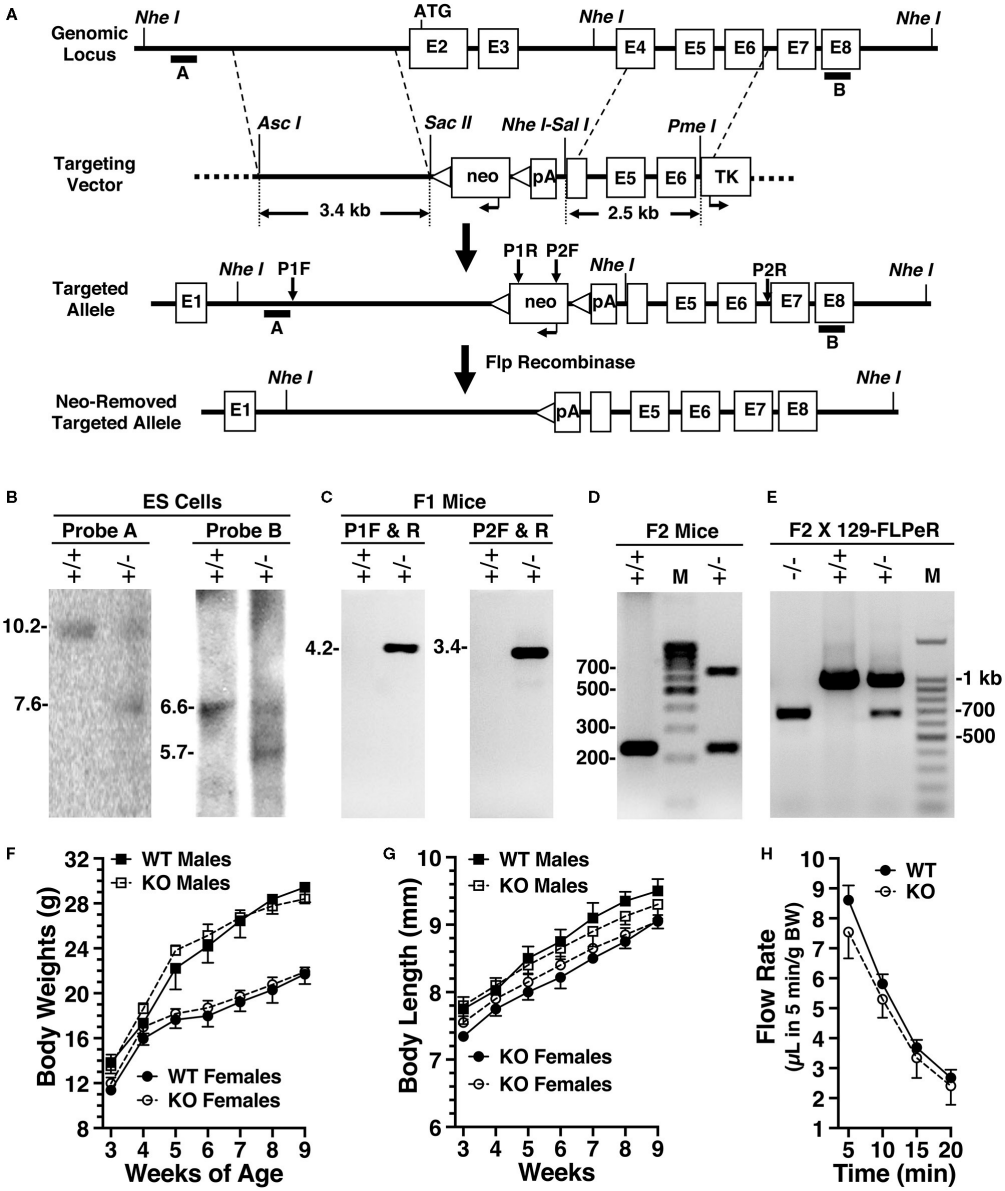
Production and initial characterizations of *Amy1* knockout mice. **(A)** The targeting vector used a base vector (pKOpA) created by the Gene Targeted Mouse Service from pBluescript II KS (Agilent Technologies, La Jolla, CA, USA). This vector incorporates a selection cassette encoding the *neo* gene under control of the herpes simplex virus–thymidine kinase promoter with two flanking FRT sites (*triangles*) and with an adjoining downstream SV40 polyA (pA). Also included is a negative selection marker, thymidine kinase, under control of the mouse phosphoglycerate kinase 1 promoter (TK). Homologous arms were PCR amplified from 129/Sv genomic DNA and cloned into pKOpA. *Asc*I and *Sac*II were used to clone the left arm; *Sal*I and *Pme*I, the right arm. Recombination excised exon 2, the translational start site, in addition to exon 3 and the 5′ portion of exon 4. Note: not drawn to scale. **(B)** Southern blot analyses of DNA from non-recombinant embryonic stem (ES) cells (+/+) and correctly targeted ES cells (+/–) digested with *Nhe*I and probed with either probe A or probe B. Probe A expected sizes are 10.2 kb (+/+) and 7.6 kb (+/–). Probe B expected sizes are 6.6 kb (+/+) and 5.7 kb (+/–). **(C)** PCR genotyping of F1 agouti mice to distinguish the absence (+/+) or presence (+/–) of germline transmission and correct insertion of the 5′- and 3′-ends of the targeted allele using primer pairs P1 and P2, as shown in **(B)**. **(D)** Genotyping before *neo* insert deletion. Expected amplicon sizes are 232 bp (+/+) and 638 bp (+/–). **(E)** PCR genotyping of male progeny for *neo* deletion after mating heterozygous F1 agouti females to a FLPeR male. Expected amplicons sizes are 998 bp (+/+) and 681 bp (+/–). **(F)** Comparisons of the body weights of male and female wild-type (WT) and *Amy1* knockout (KO) mice from 3 to 9 weeks of age. **(G)** Comparisons of the body lengths of male and female WT and *Amy1* KO mice from 3 to 9 weeks of age. **(H)** Saliva flow stimulated by subcutaneous injection of muscarinic cholinergic and β-adrenergic agonists measured at 5-min intervals after injection and the volumes normalized to body weight (BW). Results are the mean ± SE from five mice of each genotype. There are no statistical differences between the two strains of mice (two-tailed unpaired *t*-test: *P* > 0.05).

We generated *Amy1* knockout mice (*Amy1*^−*/*−^) by excising exon 2 containing the translational start site, exon 3, and the 5′ portion of exon 4, thus disrupting the expressions of both the V1 and V2 isoforms. Shown in Figures 2A–E are the targeting vector, genomic analyses of ES cell targeting, germline transmission, correct insertion of F1 agouti mice, and genotyping before and after deletion of the *neo* cassette. WT and *Amy1* KO mice were compared for a number of body characteristics; for obvious differences in internal organs; and for the number, position, size, and color of teeth, as described in *Materials and methods*. In all cases, no differences were noted between KO and WT mice (data not shown). As shown in Figures 2F–H, there were also no differences in body weight, body length, and stimulated salivary flow between WT and KO mice. In mating six pairs of heterozygous mice, the average litter size was 9, with a total of 28 males and 26 females. The genetic distribution of progeny was close to the expected: 20.4% *Amy1*^+/+^, 51.8% *Amy1*^+/−^, and 27.8% *Amy1*^−/−^. Mating three pairs of *Amy1* KO mice at 7 weeks of age produced three litters of 22 pups in total, suggesting that the litter size of KO mice was not overtly compromised.

**Figure 3:**
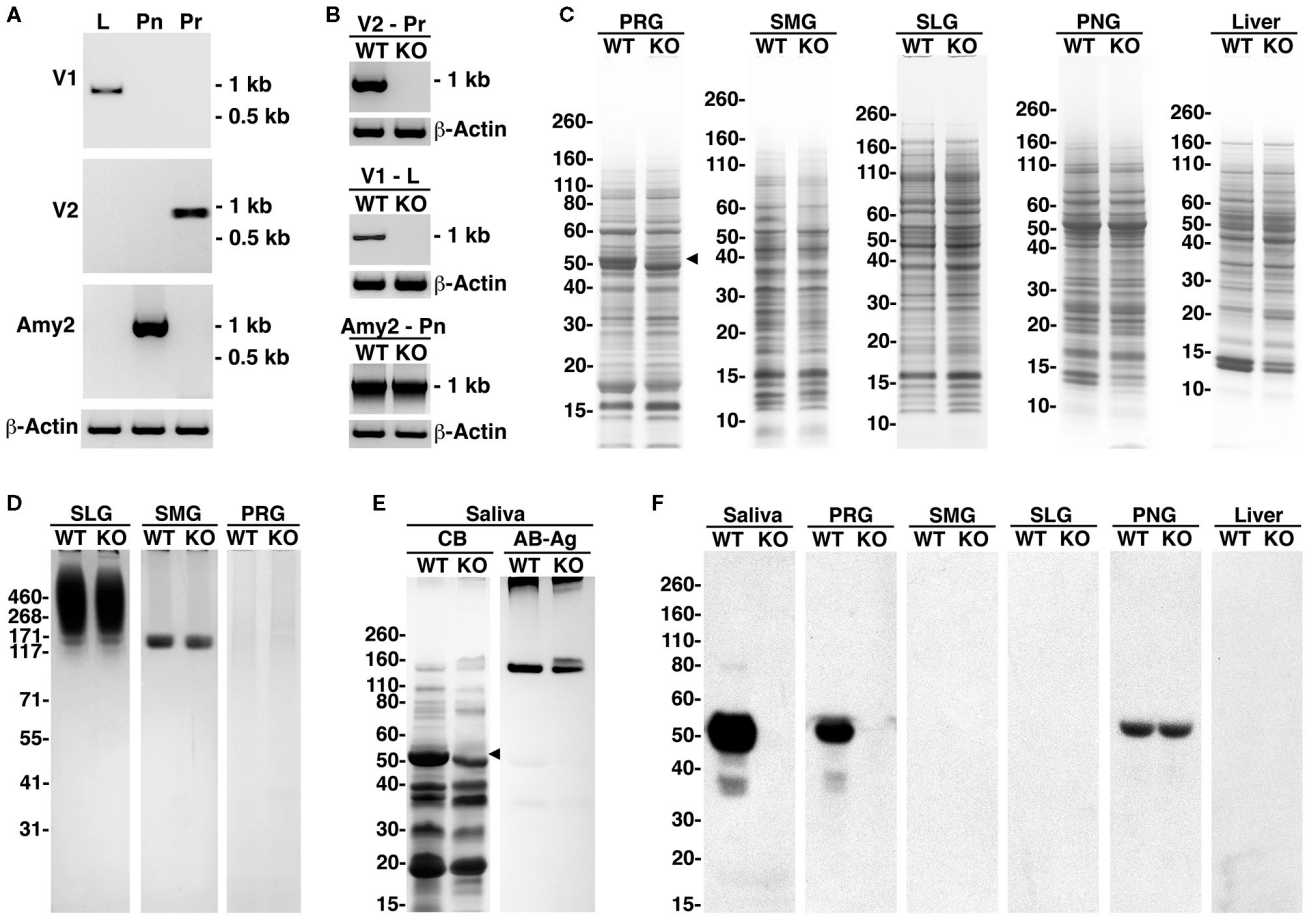
Amylase transcripts, amylase proteins, and whole tissue protein profiles of major salivary glands, pancreas, and liver and of saliva from wild-type (WT) and *Amy1* knockout (KO) mice. **(A)** Expressions in WT mice of transcripts for the V1 and V2 isoforms of *Amy1* and of *Amy2* in liver (*L*), parotid glands (*Pr*), and pancreas (*Pn*), respectively. **(B)** Expressions of amylase transcripts in Pr, L, and Pn of WT and KO mice. **(C)** Comparisons between WT and KO mice of the whole tissue protein profiles from parotid glands (*PRG*), submandibular glands (*SMG*), sublingual glands (*SLG*), pancreatic gland (*PNG*), and liver. The *black triangle* indicates an ~50-kDa band absent in the parotid glands of KO mice. SDS-PAGE 4–12% gels stained with Coomassie blue. **(D)** Expressions of highly glycosylated Muc19 mucin glycoproteins in sublingual glands and Muc10 mucins in submandibular glands were similar between WT and KO mice, whereas highly glycosylated glycoproteins were undetected in the parotid glands of mice of either genotype. SDS-PAGE 3–8% gel stained with Alcian blue and subsequent silver enhancement. **(E)** Whole stimulated saliva (6.5 μl) from WT and KO mice in an SDS-PAGE 10%−20% gradient gel stained with Coomassie blue (*CB*) or stained for glycoproteins with Alcian blue (*AB*) and subsequent enhancement with silver (*Ag*). The *black triangle* indicates an ~50-kDa protein band absent in KO saliva. **(F)** Western blots probing for amylase in saliva (15 μl) or tissue samples (200 μg wet weight) from WT and KO mice and run on 4%−12% SDS-PAGE gels.

The major salivary glands of adult mice—parotid, submandibular, and sublingual—are distinct in secretory acinar cell types and in their major secretion products. Parotid glands contain serous secretory cells with amylase as a major secretion product. Submandibular glands contain seromucous cells that produce the low-molecular-weight and non-gel-forming mucin Muc10, and sublingual glands contain mucous cells that secrete the high-molecular-weight and gel-forming mucin Muc19 (Culp et al., 2015). In probing the expressions of the amylase transcripts in WT mice, the V2 isoform of *Amy1* was found in the parotid glands, the V1 isoform in the liver, and *Amy2* in the pancreas (see Figure 3A). In comparing the expressions of the transcripts between WT and KO mice, both the V1 and V2 isoforms of *Amy1* were absent in KO mice, whereas the expression of pancreatic *Amy2* transcripts was unaltered (Figure 3B).

In examining protein expression in the parotid glands, a doublet of major proteins spanning ~48–52 kDa was observed in WT glands (Figure 3C). In KO mice, the upper band of this doublet (putative Amy1) is absent, with no other apparent differences in the protein expressions between the two genotypes. The protein profiles in the submandibular and sublingual glands between WT and KO were very similar and do not display a putative amylase band. In pancreatic and liver tissues, the protein profiles were also quite similar, with a predominant protein at ~52 kDa in pancreas, likely representing Amy2. However, a protein band at 52 kDa (putative Amy1) was not readily apparent in either WT or KO liver tissues, suggesting that *Amy1* transcripts are inefficiently translated in WT liver.

Figure 3D shows the expressions of the highly glycosylated glycoproteins in major salivary glands. Both Muc19 and Muc10 expressions in the sublingual and submandibular glands, respectively, appeared unaffected in KO mice, nor was there any aberrant mucin expression in the parotid glands. The protein and glycoprotein expressions in whole stimulated saliva from WT and KO mice are shown in Figure 3E, revealing the absence in KO mice of the upper band of the protein doublet at about 52 kDa. In this higher percentage gel, only the lower-molecular-weight components of Muc19 are observed just entering the top of the gel in saliva from mice of both genotypes. Muc10 was readily apparent in both saliva samples, but in the KO sample, two isoforms of slightly different molecular weights were expressed. The expression of these two isoforms of Muc10 is commonly observed in mice, but varies among individuals, due likely to alleles with different numbers of tandem repeats. Western blots probing for amylase in saliva and different tissues are shown in Figure 3F, demonstrating the absence of Amy1 expression in the saliva and major salivary glands of KO mice, whereas Amy2 expression in pancreatic tissue was unhindered. Amy1 protein in WT liver was not detected, consistent with a weak promoter activity and the absence of a putative Amy1 protein band in WT liver, as shown in Figure 3C. Collective evidence indicates that targeted disruption of *Amy1* expression does not alter the expression of other proteins and mucins in the saliva and salivary glands.

### Caries Development and Recovery of Bacteria

**Figure 4:**
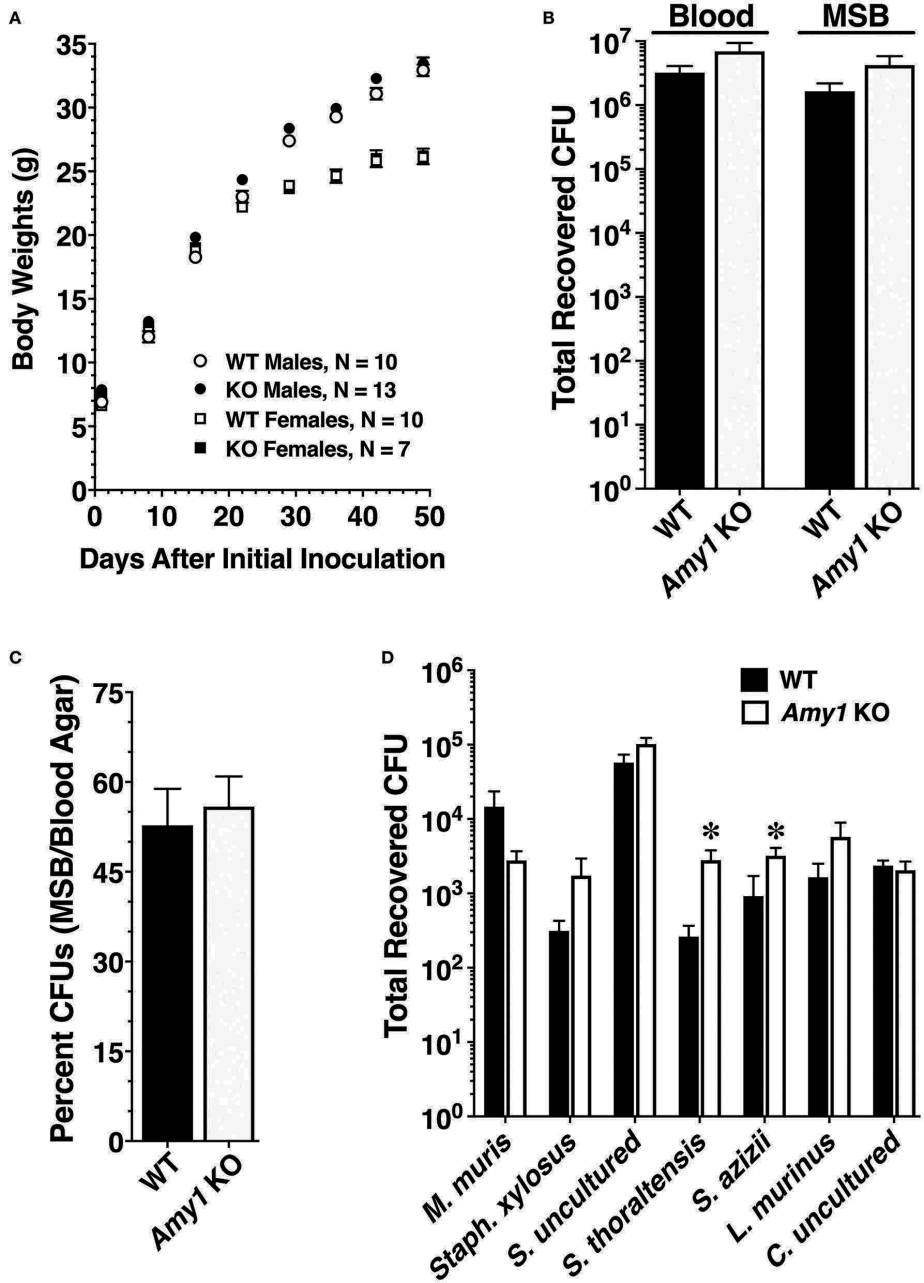
Body weights and recoveries of cultivable microbiota from wild-type (WT) and *Amy1* knockout (KO) mice in the caries experiment and comparative recoveries of autochthonous species from oral swabs. **(A)** Body weights (means ± SE) of male (*circles*) and female (*squares*) mice at weekly intervals during the caries experiment. **(B)** Recoveries of total microbiota and *S. Mutans* from sonicates of mandibular molars. Recoveries by CFUs (colony forming units) of *S. Mutans* on mitis salivarius–bacitracin (MSB) agar and total bacteria on blood agar. Values are the mean ± SE mandibular molars prepared from five pairs of mice in each group. *P* > 0.05 for KO mice compared to the WT in both cases by unpaired two-tailed *t*-test. **(C)** Expression of the data in **(B)** as the recovery of *S. mutans* (MSB agar CFUs) as a percentage of the total recovered bacteria (blood agar CFUs). Values are the mean ± SE. *P* > 0.05 for KO mice compared to the WT by unpaired two-tailed *t*-test. **(D)** Total recoveries for each of seven distinct colony morphotypes from oral swabs of each strain of mice. Oral swabs were vortexed in brain heart infusion medium and serial dilutions streaked onto duplicate blood agar plates cultured under aerobic conditions. Each colony morphotype is identified by its taxonomic species assignment from the Ribosome Database Project (RDP) database, as given in Table 2. Results are the mean ± SEM from cultures of oral swabs from 10 mice in each group. *P > 0.05 for KO mice compared to the WT by unpaired two-tailed *t*-test.

*Amy1* KO and WT mice were challenged with *S. mutans* strain UA159 and a highly cariogenic diet. Both smooth and sulcal caries were assessed. Smooth surface caries is an evaluation of the proficiency of bacteria to adhere and colonize the smooth vertical tooth surfaces, whereas sulcal caries appraises colonization within the long angular depressions of horizontal surfaces, where molars of the upper (maxilla) and lower (mandible) jaws meet and in which food particles tend to become entrapped (Barletta et al., 1988). As shown in Table 1, the mean of the incidence of total carious lesions on molar smooth surfaces (*E* score) was more than 2.3-fold higher in KO mice compared to that in WT. Furthermore, the proportional differences in caries scores further increased with each successive lesion severity score (i.e., Ds, Dm, and Dx scores), with up to 3.2-fold higher levels for the most severe type of lesion (Dx; progression into the entire dentin). The increase in smooth surface lesions occurred on all smooth surfaces of the molars (lingual, buccal, and proximal). In comparing these three sites, there was a greater proportional increase in lesions on the buccal and lingual surfaces compared to proximal surfaces. Total lesions on sulcal surfaces were nearly 1.8-fold higher in KO *vs*. WT mice. As with smooth surface scores, the proportional differences in sulcal scores increased with lesion severity to more than 5.3-fold for Dx scores. Weight gains between mice of the two genotypes during this period were very similar for each gender, an indication that all mice were healthy and consumed on average similar amounts of high-sucrose diet (Figure 4A). In examining the recoveries of bacteria from molar biofilms, there was a trend for higher recoveries from KO mice *vs*. WT mice of total cultivable bacteria from cultures on blood agar and of *S. mutans* from MSB agar. However, these differences were not significantly different (Figure 4B). Recovered *S. mutans* accounted for more than 50% of the estimated total recovered bacteria from both WT and KO mice, with a trend toward a higher percentage recovered from KO mice (Figure 4C).

**Table 1:** Development of caries and their severities on molars comparing wild-type mice with *Amy1* knockout (KO) mice.

	**Wild type**	***Amy1* KO**
Smooth surfaces		
Total *E*	9.35 (1.14)	21.95 (2.53)^*^
Total Ds	5.95 (0.87)	16.65 (2.48)^*^
Total Dm	4.10 (0.81)	12.50 (1.96)^*^
Total Dx	3.60 (0.83)	11.60 (1.87)^*^
Buccal *E*	2.00 (0.45)	5.70 (0.73)^*^
Buccal Ds	0.90 (0.34)	2.85 (0.70)^*^
Buccal Dm	0.45 (0.26)	2.25 (0.55)^*^
Buccal Dx	0.40 (0.26)	1.85 (0.49)^*^
Lingual *E*	3.80 (0.58)	9.90 (1.45)^*^
Lingual Ds	2.45 (0.54)	8.20 (1.35)^*^
Lingual Dm	2.15 (0.49)	6.65 (1.12)^*^
Lingual Dx	1.90 (0.46)	6.30 (1.08)^*^
Proximal *E*	3.55 (0.52)	6.35 (0.65)^*^
Proximal Ds	2.60 (0.41)	5.60 (0.65)^*^
Proximal Dm	1.50 (0.37)	3.60 (0.57)^*^
Proximal Dx	1.30 (0.38)	3.45 (0.56)^*^
Sulcal surfaces		
Total *E*	9.85 (1.11)	17.65 (1.86)
Total Ds	6.95 (1.08)	15.20 (1.71)^*^
Total Dm	4.20 (0.78)	10.95 (1.45)^*^
Total Dx	1.60 (0.49)	8.50 (1.33)^*^

*Values are the mean (SE) of Larson's modified Keyes' scores (Larson, 1981). 20 mice per group. Total smooth surface caries is the sum of buccal, lingual, and proximal caries, representing surfaces juxtaposed to the cheeks, tongue, and an adjacent tooth, respectively. Severity scores are: E (enamel affected), Ds (dentin exposed), Dm (3/4 of the dentin affected), and Dx (whole dentin affected). Comparisons are by ANOVA with Tukey–Kramer post-hoc test at 95% and 90% confidence.*

* *P ≤ 0.05 vs. WT; P ≤ 0.10 vs. WT*.

### The Oral Microbiome and Changes in the Absence of Amylase Expression

An examination of whether the deletion of Amy1 expression affects the composition of indigenous oral microbiota was undertaken. We previously demonstrated no differences in recovered bacteria when culturing bacteria from oral swabs of WT and Muc19 KO mice under anerobic or aerobic (95% air−5% CO_2_) conditions (Culp et al., 2015). Hence, oral swabs from uninfected WT and KO mice were streaked on blood agar and cultured aerobically. Seven identical colony morphotypes were recovered consistently from mice of both genotypes, and these morphotypes were identical to those isolated from mice in our colonies described previously (Culp et al., 2015). No colonies formed when the swabs were streaked on MSB agar, indicating the absence of indigenous mutans streptococci. Also, no additional colony morphologies were apparent with extended culture on blood agar. Shown in Table 2 are the characteristics of each colony morphotype and their taxonomic assignments based on alignments of consensus 16S ribosomal RNA (rRNA) sequences to the Ribosome Database Project. Consensus sequences ranged from 1,427 to 1,469 bp. Note that the colony morphotypes and their 16S rRNA sequences were identical between WT and KO mice. The sequences aligned with identities of 99.1–100% to cultured or uncultured bacteria isolated from mice. For comparisons to human oral microbiota, each consensus sequence is also aligned to the annotated HOMD 16S rRNA reference sequences from the human oral microbiome. Note that the 16S rRNA sequences of murine oral streptococcal species most closely aligned in the HOMD database to oral commensal streptococci commonly associated with oral health. It should also be mentioned that the 16S rRNA sequences from each colony morphotype were identical to those reported previously in our colonies of WT and Muc19 KO mice, although some species classifications have changed due to the expansion of sequences within the Ribosome Database Project (Culp et al., 2015). Thus, our colonies of mice had identical oral commensals, which is not surprising as they were derived using the same FLPeR male and Black Swiss and NFS/N mice from the same in-house and access-restricted colonies.

**Table 2:** Phenotypic characteristics and 16S rRNA sequence alignments of colonies isolated on blood agar from oral swabs of wild-type and *Amy1* knockout mice under aerobic conditions.

**Colony and cell appearances**	**Gram stain**	**Catalase activity**	**Reference sequences**	**Genus**	**Species**	**GenBank accession**	**Percent identity**
Large gray, rods	–	+	RDP	*Muribacter*	*muris*	KP278132	99.9
			HOMD	*Haemophilus*	*haemolyticus*	HM596277	93.9
Large yellow, cocci	+	+	RDP	*Staphylococcus*	*xylosus*	D83374	99.9
			HOMD	*Staphylococcus*	*cohnii*	NR_036902	98.8
Gray α-hemolytic, cocci	+	–	RDP	*Streptococcus*	*uncultured*	EU534703	99.9
			HOMD	*Streptococcus*	*sanguinis*	AF003928	96.6
Irregular large gray, cocci	+	–	RDP	*Streptococcus*	*thoraltensis*	Y09007	99.1
			HOMD	*Streptococcus*	*salivarius*	M58839	94.3
White, cocci	+	–	RDP	*Streptococcus*	*azizii*	KM609123	100
			HOMD	*Streptococcus*	*cristatus*	AB008313	95.3
White, rods	+	–	RDP	*Lactobacillus*	*murinus*	AF157049.1	99.8
			HOMD	*Lactobacillus*	*salivarius*	AF089108	94.1
Small gray, rods	+	+	RDP	*Corynebacterium*	Uncultured	FJ892757^a^	99.5
			HOMD	*Corynebacterium*	*mastitidis*	NR_026376	98.4

*Cell appearance was determined under light microscopy (×100). High-quality consensus 16S rRNA sequences from colony PCR of single isolates from three mice of each genotype were aligned by the BLAST server at the Human Oral Microbiome Database (HOMD; www.homd.org/index.php) to two sets of 16S rRNA reference sequences, HOMD 16S rRNA RefSeq 15.1 (Chen et al., 2010) and sequences of the Ribosome Database Project (RDP), release 11.5 (Cole et al., 2014). The highest scoring alignments were identical between consensus sequences from wild-type and Amy1 knockout mice. Percent identities are the total nucleotide mismatches per total matching nucleotides, including gaps*.

The recoveries of each colony morphotype were compared between WT and KO mice (Figure 4D). There were no differences in the total recovered CFUs between the two genotypes (WT: means ± SE = 7.76 × 10^4^ ± 2.07 × 10^4^; KO: means ± SE = 1.20 × 10^5^ ± 2.12 × 10^4^; *n* = 10, *P* = 0.168, unpaired two-tailed *t*-test). However, in the absence of salivary Amy1, there was a significant increase in the relative proportions of *Streptococcus thoraltensis* (>10-fold) and *Streptococcus azizii* (78%) (Figure 4D). There was also a trend for increased *Lactobacillus murinus* and *Staphylococcus xylosus* and decreased *Muribacter muris* in KO mice. Collectively, deletion of Amy1 expression increased the dominance of streptococcal species in the oral cavity from ~75 to 90%.

### *In vitro* Assessments of Potential Protective Mechanisms

**Figure 5:**
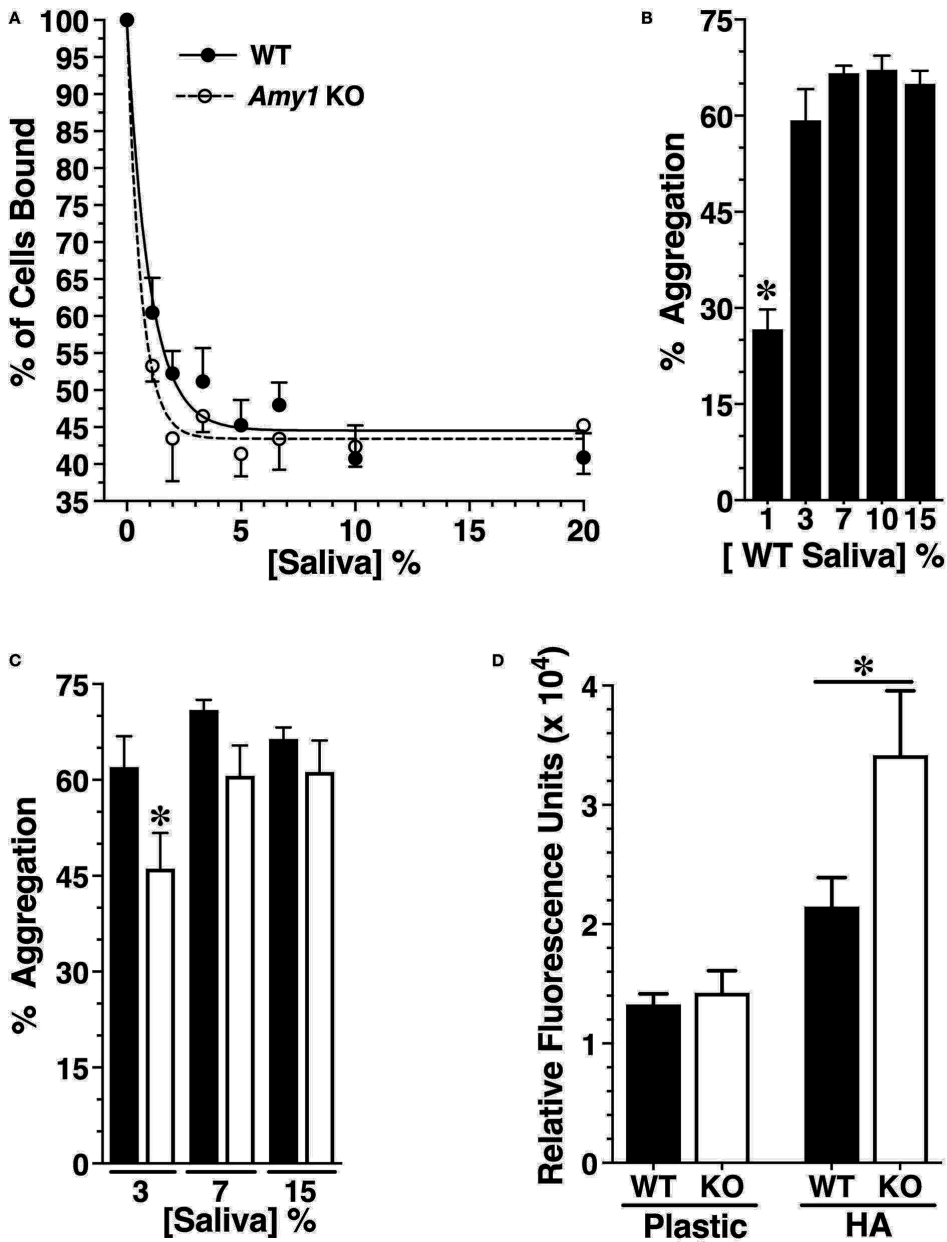
Comparing innate immune functions *in vitro* between whole stimulated saliva from wild-type (WT) and *Amy1* knockout (KO) mice. **(A)** Adherence of *S. mutans* to hydroxyapatite discs pretreated with increasing concentrations of whole stimulated saliva from WT and KO mice. Adherence is normalized to the percent of cells bound in the absence of saliva. Values are mean ± SE from five separate experiments. *P* > 0.05 for all comparisons between WT and KO at each concentration of saliva. **(B)** Percent aggregation of *S. mutans* UA159 with increasing concentrations of whole stimulated saliva from WT mice. Values are the mean ± SE from 10 separate experiments. *P < 0.002 *vs*. all higher concentrations of saliva. **(C)** Aggregation of *S. mutans* with increasing concentrations of whole stimulated saliva from WT and KO mice. Values are the mean ± SE from 16 separate experiments. *P < 0.05 *vs*. WT. **(D)** *S. mutans* UA159 within biofilms cultured 24 h under aerobic conditions on plastic or on hydroxyapatite discs (HA) in semi-defined biofilm medium containing 1% glucose and supplemented without or with 25% (*v*/*v*) whole stimulated saliva from KO or WT mice. After washing to remove planktonic and loosely bound cells, *S. mutans* were quantified by fluorescent staining of DNA with SYTO 13. Values are the mean ± SE from seven separate experiments. *P < 0.033. *P* < 0.05 for conditions on plastic *vs*. conditions on HA. All comparisons are by one-way ANOVA with Tukey's multiple comparisons test.

To interrogate the possible mechanisms through which Amy1 may limit the cariogenicity of *S. mutans*, a series of *in vitro* assays were conducted comparing the interaction of *S. mutans* with whole stimulated and non-clarified saliva from WT and KO mice under three different conditions: adherence to an acquired salivary pellicle, aggregation in solution, and development of a biofilm. Our rationale for conducting assays containing glucose as the only carbohydrate source was to allow assessments of the influence of amylase on limiting dental colonization by *S. mutans* under conditions representing oral health rather than under cariogenic conditions in which adherence and biofilm growth are overshadowed by the influence of glucosyltransferases and extracellular glucan polysaccharides (Koo et al., 2010). Shown in Figure 5A is the adherence of *S. mutans* to hydroxyapatite pretreated with increasing concentrations of saliva from WT and KO mice to produce salivary pellicles. *S. mutans* binds avidly to naked hydroxyapatite (Vacca-Smith et al., 1994), whereas the formation of an overlying salivary pellicle limits adherence to sites formed by specific salivary constituents (Clark et al., 1978). We found that the adherence of *S. mutans* was increasingly inhibited with progressively higher concentrations of saliva, reaching a steady state of ~55% inhibition compared to naked hydroxyapatite at about 5% saliva. There were no significant differences between saliva from WT *vs*. KO mice, suggesting that the absence of salivary amylase does not alter the binding sites for *S. mutans* in salivary pellicles.

When in solution, the constituents of saliva promote bacterial aggregation and subsequent clearance from the oral cavity upon swallowing. We therefore examined whether Amy1 in saliva functions to promote the aggregation of *S. mutans*. As little as 3% saliva from WT mice was sufficient for maximal aggregation of *S. mutans* (Figure 5B). In comparing WT and KO saliva samples, there was a very modest decrease in aggregation with 3% saliva from KO mice *vs*. WT mice, with no significant differences at higher concentrations (Figure 5C). The results suggest that amylase has only a very minor role in the aggregation and clearance of *S. mutans*.

The saliva samples were next compared for their impact on *S. mutans* biofilm growth in a semi-defined medium containing 1% glucose, when cultured on either polystyrene or on hydroxyapatite after allowing for the formation of a salivary pellicle. *S. mutans* within biofilms was assessed by the incorporation of the DNA-binding fluorescent dye SYTO 13. As shown in Figure 5D, when cultured on a hydrophilic polystyrene surface, there were no differences in the SYTO 13 signal when in the presence of saliva with or without amylase. However, when cultured onto hydroxyapatite in the presence of KO saliva, *S. mutans* within biofilms was ~40% less when in WT saliva *vs*. KO saliva.

## Discussion

Mice with targeted deletion of Amy1 were generated and demonstrated the absence of Amy1 in the parotid glands and saliva without affecting Amy2 expression in the pancreas. Although *Amy1* mRNA in the liver of KO mice was undetectable, the absence of Amy1 protein in WT liver suggests that its mRNA is inefficiently translated and the resultant proteins secreted into blood, where it is found at very low levels (Koyama et al., 2001).

### Amylase, Caries, and Diet

The results from the caries experiment clearly demonstrate that the amylase in saliva has marked protective effects against both sulcal and smooth surface caries. It should be noted that mice were fed a diet containing 6% whole wheat flour and 4% alfalfa meal that together supplied ~5% starch to the total diet. In a study with conventional rats allowed to feed on diets containing 5% brewer's yeast supplemented with either 95% sucrose, 95% wheat starch, or 47.5% sucrose plus 47.5% wheat starch, and other nutrients administered *via* gastric gavage, there were no differences in the smooth surface and sulcal caries induced by *S. mutans* between those fed only sucrose compared to sucrose plus starch, although rats fed only starch had significantly less caries than the rats fed either of the two other diets (Firestone et al., 1982). In a study with gnotobiotic rats fed a diet containing 1% sucrose and 66% corn starch and challenged with *S. mutans*, the caries levels were very near maximal levels that were induced when mice were fed diets containing 3 or 10% sucrose, where cornstarch was replaced by sucrose in each diet (Michalek et al., 1977). Collective results are consistent with dietary starch and sucrose working in a cooperative manner to promote caries induced by *S. mutans*, but that maximal caries occurs over broad ranges of sucrose and starch. It may be that a limited amount of dietary starch is sufficient to saturate retentive sites on teeth to promote the initiation and progression of carious lesions induced by underlying *S. mutans* in the presence of sucrose. Hence, the use of a diet higher in starch may not have a dramatic impact on caries induced in WT mice. However, with KO mice, it is unclear whether increasing dietary starch would result in even higher levels of caries. We acknowledge that Diet 2000, commonly used in rodent caries experiments, is highly cariogenic, but its use is designed to induce high levels of caries to better detect statistically small differences between animal groups, especially when it is unclear whether the experimental condition to be tested will have any effect on caries incidence and severities. This same reasoning is behind the use of a relatively large number of animals per group. At the same time, this dietary regimen represents a highly stringent test of the innate protective mechanisms associated with the oral cavity and, in particular, its hard tissues. Our rationale for using *S. mutans* UA159 is that it is widely used as a model in caries research, both in *in vitro* and animal studies, in which the genetic elements responsible for its colonization and virulence are being deciphered to better understand its pathogenesis and delineate potential therapeutic targets against mutans streptococci (Klein et al., 2010; Bowen et al., 2018; Ahn et al., 2020; Culp et al., 2021). The fact that the deletion of amylase expression resulted in a marked increase in caries answers our original question of whether its overriding effect is to either help promote or protect against caries. It is therefore anticipated that this result will invite further *in vitro*, animal, and human studies by the research community that will be directed toward the beneficial properties of this major salivary component in defending dental surfaces.

### Amylase Expression in Humans and Caries

In the context of human caries, our results are consistent with a study that reported higher salivary amylase activity in caries-free children compared to children with early childhood caries (Borghi et al., 2017). Also, in a pilot study, amylase associated with *in situ* acquired enamel pellicles of children in a caries-active group was half that of a caries-free group, although the difference was not significant, likely due to the small sample size with large variabilities (Hertel et al., 2019). In contrast, a positive correlation was reported between the level of salivary amylase and the number of decayed, missing, and filled teeth among 32 male adults (Vitorino et al., 2006). In humans, the copy number of *AMY1* genes can vary from 2 to 24, with the *AMY1* copy number correlated positively with the level of salivary amylase protein and enzymatic activity (Boehlke et al., 2015; Poole et al., 2019). Interestingly, the mean gene copy number of *AMY1* correlates positively with the level of dietary starch consumed by different human populations, with a pronounced higher mean copy number in agricultural societies compared to hunter-gatherer societies (Perry et al., 2007). *AMY1* gene duplications are thought to result from selective pressures by greater consumption and energy utilization from starch during the agricultural revolution (Fried et al., 1987; Perry et al., 2007; Iskow et al., 2012). Coinciding with the onset of human agriculture is the exponential expansion of the population of *S. mutans*, with the addition to its core genome of genes associated with virulence properties (i.e., sugar metabolism and acid tolerance) (Cornejo et al., 2013) that further coincides with the increased caries prevalence in humans (Boehlke et al., 2015). Selective pressure for increased *AMY1* in protection against caries may therefore have contributed to the increased *AMY1* copy number, as edentulism leads to higher physical and psychological disability and subsequent mortality (Emami et al., 2013).

### Murine Oral Commensals and the Influence of Amylase

Non-mutans streptococci represented the predominant commensals of the microbiota from oral swabs of WT and KO mice, consistent with previous observations in humans (Lazarevic et al., 2009) as well as laboratory mice (Chun et al., 2010). *S. thoraltensis* was isolated previously from human subgingival plaque of patients with periodontitis (Dhotre et al., 2016). *S. azizii* was earlier isolated from the oral cavity of inbred C57BL mice (Braden et al., 2015) and the uncultured species of streptococcus was found in the epidermis of mice, likely transferred orally during grooming (Grice et al., 2008). Other oral commensals have also been identified in mice, including uncultured corynebacterium (Scharschmidt et al., 2009), *S. xylosus* (Gozalo et al., 2010), and *L. murinus* (Almiron et al., 2013). *M. muris* was originally classified as *Actinobacillus muris* (Bisgaard, 1986) and is commonly found colonizing murine mucosal surfaces (Kahl et al., 2020). The absence of Amy1 expression promoted the oral colonization of *S. thoraltensis* and *S. azizii* with a trend for increased *L. murinus* and *S. xylosus* and the attenuation of *M. muris* colonization, thus highlighting the impact of a single salivary constituent on the oral microbiota. In a study with human subjects with either a high or low *Amy1* gene copy number and provided a standardized diet, those with a low copy number exhibited an oral microbiota with a higher abundance of members of the genera *Prevotella* and *Porphyromonas* and lesser abundance of *Haemophilus* and *Neisseria* (Poole et al., 2019), further demonstrating that salivary amylase impacts the composition of the oral microbiota.

Because amylase expression did not impact *S. mutans* colonization of dental plaque, we speculate that the protective effect of amylase against caries resulted from mechanisms that attenuated plaque acidity to decrease enamel demineralization, as manifested by the markedly less incidence of caries and their severities. Many Gram-positive and Gram-negative bacteria can convert arginine to ornithine, ammonia, CO_2_, and ATP *via* an arginine deiminase system (ADS), including lactobacilli (Zuniga et al., 1998), staphylococci (Lindgren et al., 2014), and many species of oral streptococci (Velsko et al., 2018). Generation of ammonia within biofilms can provide less acid-tolerant commensal bacteria with the means to better persist when in competition with the acid-generating and more acid-tolerant *S. mutans* (Huang et al., 2015). Moreover, accruing clinical data are consistent with the attenuation of dental caries and changes in oral ecology associated with higher levels of ammonia generation within dental plaque (Peterson et al., 1985; Shu et al., 2007; Nascimento et al., 2009, 2013). ADS activity is induced by arginine, but can undergo strong or weak repression by carbon catabolite repression, in which one carbohydrate is preferred over others that are available (Zuniga et al., 1998; Liu et al., 2008). The reported results have shown that the ADS of oral streptococci (Dong et al., 2004; Liu et al., 2008), different species of lactobacilli (Zuniga et al., 1998), and staphylococci (Lindgren et al., 2014) can undergo either stronger or weaker carbon catabolite repression by glucose compared to galactose. Thus, it is reasonable to posit that one or more commensals within dental biofilms of mice expressed high ADS activity and that, with a lower availability of glucose from amylase hydrolysis of starch, the ADS activity was more repressed by the alternate carbohydrates used for energy, thus exposing dental enamel to more acidic conditions. Alternatively, because the oral microbiota was altered by the deletion of amylase expression, the colonization of dental biofilms by commensals with high ADS activity and/or commensals expressing ADS that favor glucose may have been decreased. Because amylase is considered to promote dental colonization by commensals expressing an ABP, it would be of interest in future studies to evaluate the expression of ABPs and the ADS activities of oral commensal species and to correlate their abundance in dental biofilms as a function of amylase expression.

Another explanation to consider for increased caries with deletion of amylase expression is that caries resulted from greater oral colonization by *S. thoraltensis* and/or *S. azizii*, assuming they are significantly aciduric and acidogenic, although based on the 16S rRNA sequences they are most closely related to the human commensals *Streptococcus salivarius* and *Streptococcus cristatus*, respectively. We have recently shown that oral swabs did not extract significant amounts of bacteria from dental plaque, but instead primarily extracted bacteria from the mucosal pellicle and saliva (Culp et al., 2021). Thus, it is unclear whether there were any significant differences in the colonization of dental biofilms by specific commensal species. Furthermore, *S. mutans* accounted for >50% of the total recovered bacteria from dental plaque of both WT and KO mice, indicating no significant differences in dental colonization by commensals as a whole. We have also recently shown that murine oral commensals did not produce any significant caries when mice were fed a highly cariogenic diet (Ahn et al., 2020). Although we cannot discount commensal streptococci accounting for a portion of the increased caries, it is unlikely that they would have accounted for a great majority of the observed difference. Nevertheless, future studies may assess the abundance in dental biofilms of each commensal species as a function of amylase expression and test their aciduric and acidogenic characteristics. Alternatively, amylase KO mice could be tested for caries development under highly cariogenic conditions, with and without *S. mutans* challenge.

### Influence of Amylase on *S. mutans in vitro*

The similar recoveries of *S. mutans* from dental biofilms between WT and KO mice are consistent with *in vitro* assays where the expression of amylase in saliva did not substantially alter processes that can affect *S. mutans* colonization (i.e., adherence to saliva-coated hydroxyapatite and aggregation) and further indicate that *S. mutans* does not bind to amylase to any appreciable extent. Interestingly, the expression of salivary amylase reduced *S. mutans* within biofilms on hydroxyapatite by ~40%, when in the presence of whole saliva and with glucose as the sole carbohydrate. However, the negative effect of salivary amylase on *S. mutans* within biofilms was not present with biofilms formed on polystyrene, suggesting a requirement for amylase within an acquired enamel pellicle formed on hydroxyapatite for hindrance of biofilm formation. Furthermore, amylase protein rather than its enzymatic activity appears necessary for the observed inhibition as the biofilms were formed in the absence of starch. Because current and previous evidence (Nikitkova et al., 2013) indicate that *S. mutans* does not interact with amylase directly, it may recognize an epitope within a complex of amylase and another salivary constituent, in which the epitopes are partially masked by amylase when the complex is bound to hydroxyapatite. In the case of polystyrene as a substrate, such amylase complexes may not bind and form part of the pellicle. Interestingly, when cultured on a salivary pellicle on hydroxyapatite in the presence of starch and sucrose, biofilms of *S. mutans* displayed a lower cell volume compared to the biofilms cultured in sucrose alone (Klein et al., 2009). Moreover, the production of extracellular polysaccharides that are expected to promote *S. mutans* colonization was increased (Klein et al., 2009). These changes were correlated with complex alterations in the expressions of genes associated with sugar metabolism, environmental stress, and two-component systems and attributed primarily to the availability of starch hydrolysates from the action of amylase (Klein et al., 2010). Thus, in monospecies biofilms, *in vitro*, the presence of amylase protein within a salivary pellicle and amylase hydrolysis of starch both appear to negatively impact *S. mutans* colonization. These collective results suggest that amylase serves to limit the growth of *S. mutans* on salivary pellicles initially formed on clean enamel surfaces. Nevertheless, when within the more complex *in vivo* environment that includes oral commensals and a cariogenic diet, the negative effect of amylase on *S. mutans* biofilm colonization appears overshadowed as the expression of salivary amylase did not affect the recovery of *S. mutans* from the dental biofilms of KO mice compared to WT mice.

As described in Materials and Methods, the UA159 strain used in the current study encodes a non-functional, truncated PerR. It has long been in use in many laboratories, has enhanced tolerance to oxidative stress (Kajfasz et al., 2021), and, compared to the genetically identical strain except for a single deletion in the *perR* gene, displays more competitiveness *in vitro* against H_2_O_2_-producing human oral commensals (Burne, personal communication). The *perR* mutation is therefore not expected to have diminished the ability of this strain to colonize mice and induce caries. However, we suggest that other strains of *S. mutans* be evaluated in the future, especially recent clinical isolates from carious lesions of humans that express high *vs*. low levels of amylase, as these strains may behave differently after being adapted to environments with different levels of not only amylase but possibly also dissimilar oral microbiomes and levels of dietary starch.

## Conclusion

The current study demonstrates that salivary amylase plays a role in the protection of dental surfaces from caries induced by *S. mutans* under highly cariogenic conditions, thus supporting the limited clinical studies described above that suggest a protective role for amylase. Further examinations of the role of amylase in the caries process are warranted, including the impact of amylase on the composition of the microbiota of dental biofilms and the mechanisms that may attenuate plaque acidity. Moreover, future studies of biomarkers for caries risk (Lorenzo-Pouso et al., 2018; Wang et al., 2019) should include levels of salivary amylase protein and enzymatic activity, especially in predicting the risk of early childhood caries (Borghi et al., 2017).

## Data Availability Statement

The raw data supporting the conclusions of this article will be made available by the authors, without undue reservation.

## Ethics Statement

The animal study was reviewed and approved by The University of Florida IACUC Committee.

## Author Contributions

DC was responsible for the conception and experimental design of the study, data analysis, and interpretation of the results and drafted and critically revised the manuscript. MC was responsible for all work to verify correct insertion of the targeting vector, tissue expression of amylase mRNA, glandular and saliva expression of proteins, glycoproteins and amylase protein, and for all *in vitro* assays. BR was responsible for genotyping mice, managing mouse colonies, and gross phenotypic characterization of mice, conducted the caries experiment, and performed caries scoring after undergoing calibration under the direction of DC and also responsible for enumeration of the recovered bacteria and 16S rRNA sequencing. All authors have read and approved the submitted manuscript.

## Conflict of Interest

The authors declare that the research was conducted in the absence of any commercial or financial relationships that could be construed as a potential conflict of interest.
