# Supplementary material for: Murine Salivary Amylase Protects Against Streptococcus mutans-Induced Caries
Source: Front Physiol. 2021 Jul 2;12:699104. doi: 10.3389/fphys.2021.699104 (PMC8283412; doi:10.3389/fphys.2021.699104)
Supplement: Supplementary file 1 [file Image_1.PDF]

```
                  10        20        30        40        50        60        70        80        90       100
Amy1 V1   MKFFLLLSLIGFCWAQYDPHTQYGRTAIVHLFEWRWVDIAKECERYLAPNGFAGVQVSPPNENIVVHSPSRPWWERYQPISYKICSRSGNEDEFRDMVNR
Amy1 V2   MKFFLLLSLIGFCWAQYDPHTQYGRTAIVHLFEWRWVDIAKECERYLAPNGFAGVQVSPPNENIVVHSPSRPWWERYQPISYKICSRSGNEDEFRDMVNR
Amy2a1    MKFVLLLSLIGFCWAQYDPHTSDGRTAIVHLFEWRWVDIAKECERYLAPKGFGGVQVSPPNENVVVHNPSRPWWERYQPISYKICTRSGNEDEFRDMVTR
Amy2a2    MKFVLLLSLIGFCWAQYDPHTSDGRTAIVHLFEWRWVDIAKECERYLAPKGFGGVQVSPPNENVVVHNPSRPWWERYQPISYKICTRSGNEDEFRDMVTR
Amy2a3    MKFVLLLSLIGFCWAQYDPHTSDGRTAIVHLFEWRWVDIAKECERYLAPKGFGGVQVSPPNENVVVHNPSRPWWERYQPISYKICTRSGNEDEFRDMVTR
Amy2a4    MKFVLLLSLIGFCWAQYDPHTSDGRTAIVHLFEWRWVDIAKECERYLAPKGFGGVQVSPPNENVVVHNPSRPWWERYQPISYKICTRSGNEDEFRDMVTR
Amy2a5    MKFVLLLSLIGFCWAQYDPHTSDGRTAIVHLFEWRWVDIAKECERYLAPKGFGGVQVSPPNENVVVHNPSRPWWERYQPISYKICTRSGNEDEFRDMVTR
Amy2b     MKFVLLLSLIGFCWAQYDPHTSDGRTAIVHLFEWRWVDIAKECERYLAPKGFGGVQVSPPNENIVIHNPSRPWWERYQPISYKICTRSGNEDEFRDMVTR

                 110       120       130       140       150       160       170       180       190       200
Amy1 V1   CNNVGVRIYVDAVINHMCGVGAQAGQSSTCGSYFNPNNRDFPGVPYSGFDFNDGKCRTASGGIENYQDAAQVRDCRLSGLLDLALEKDYVRTKVADYMNH
Amy1 V2   CNNVGVRIYVDAVINHMCGVGAQAGQSSTCGSYFNPNNRDFPGVPYSGFDFNDGKCRTASGGIENYQDAAQVRDCRLSGLLDLALEKDYVRTKVADYMNH
Amy2a1    CNNVGVRIYVDAVINHMCGAGNPAGTSSTCGSYLNPNNREFPAVPYSAWDFNDNKCN---GEIDNYNDAYQVRNCRLTGLLDLALEKDYVRTKVADYMNH
Amy2a2    CNNVGVRIYVDAVINHMCGAGNPAGTSSTCGSYLNPNNREFPAVPYSAWDFNDNKCN---GEIDNYNDAYQVRNCRLTGLLDLALEKDYVRTKVADYMNH
Amy2a3    CNNVGVRIYVDAVINHMCGAGNPAGTSSTCGSYLNPNNREFPAVPYSAWDFNDNKCN---GEIDNYNDAYQVRNCRLTGLLDLALEKDYVRTKVADYMNH
Amy2a4    CNNVGVRIYVDAVINHMCGAGNPAGTSSTCGSYLNPNNREFPAVPYSAWDFNDNKCN---GEIDNYNDAYQVRNCRLTGLLDLALEKDYVRTKVADYMNH
Amy2a5    CNNVGVRIYVDAVINHMCGAGNPAGTSSTCGSYLNPNNREFPAVPYSAWDFNDNKCN---GEIDNYNDAYQVRNCRLTGLLDLALEKDYVRTKVADYMNH
Amy2b     CNNVGVRIYVDAVINHMCGSGNPAGTSSTCGSYLNPNNREFPAVPYSAWDFNDNKCN---GEISNYNDAYQVRNCRLSGLLDLALEKDYVRTK-------

                 210       220       230       240       250       260       270       280       290       300
Amy1 V1   LIDIGVAGFRLDASKHMWPGDIKAILDKLHNLNTKWFSQGSRPFIFQEVIDLGGEAVSSNEYFGNGRVTEFKYGAKLGKVMRKWDGEKMSYLKNWGEGWG
Amy1 V2   LIDIGVAGFRLDASKHMWPGDIKAILDKLHNLNTKWFSQGSRPFIFQEVIDLGGEAVSSNEYFGNGRVTEFKYGAKLGKVMRKWDGEKMSYLKNWGEGWG
Amy2a1    LIDIGVAGFRLDAAKHMWPGDIKAVLDKLHNLNTKWFSQGSRPFIFQEVDQYIIFKILILLFNYSVSSFPFIPPHTSMYLSAVT
Amy2a2    LIDIGVAGFRLDAAKHMWPGDIKAVLDKLHNLNTKWFSQGSRPFIFQEVIDLGGEAIKGSEYFGNGRVTEFKYGAKLGTVIRKWNGEKMSYLKNWGEGWG
Amy2a3    LIDIGVAGFRLDAAKHMWPGDIKAVLDKLHNLNTKWFSQGSRPFIFQEVIDLGGEAIKGSEYFGNGRVTEFKYGAKLGTVIRKWNGEKMSYLKNWGEGWG
Amy2a4    LIDIGVAGFRLDAAKHMWPGDIKAVLDKLHNLNTKWFSQGSRPFIFQEVIDLGGEAIKGSEYFGNGRVTEFKYGAKLGTVIRKWNGEKMSYLKNWGEGWG
Amy2a5    LIDIGVAGFRLDAAKHMWPGDIKAVLDKLHNLNTKWFSQGSRPFIFQEVIDLGGEAIKGSEYFGNGRVTEFKYGAKLGTVIRKWNGEKMSYLKNWGEGWG
Amy2b     ------------------------------------------------VIDLGGEAIKGSEYFGNGRVTEFKFGAKLGTVIRKWNGEKMSYLKNWGEGWG

                 310       320       330       340       350       360       370       380       390       400
Amy1 V1   LMPSDRALVFVDNHDNQRGHGAGGASILTFWDARLYKMAVGFMLAHPYGFTRVMSSYYWPRNFQNGKDVNDWVGPPNNNGKTKEVSINPDSTCGNDWICE
Amy1 V2   LMPSDRALVFVDNHDNQRGHGAGGASILTFWDARLYKMAVGFMLAHPYGFTRVMSSYYWPRNFQNGKDVNDWVGPPNNNGKTKEVSINPDSTCGNDWICE
Amy2a1
Amy2a2    LVPSDRALVFVDNHDNQRGHGAGGSSILTFWDARMYKMAVGFMLAHPYGFTRVMSSYRWNRNFQNGKDQNDWIGPPNNNGVTKEVTINADTTCGNDWVCE
Amy2a3    LVPSDRALVFVDNHDNQRGHGAGGSSILTFWDARMYKMAVGFMLAHPYGFTRVMSSYRWNRNFQNGKDQNDWIGPPNNNGVTKEVTINADTTCGNDWVCE
Amy2a4    LVPSDRALVFVDNHDNQRGHGAGGSSILTFWDARMYKMAVGFMLAHPYGFTRVMSSYRWNRNFQNGKDQNDWIGPPNNNGVTKEVTINADTTCGNDWVCE
Amy2a5    LVPSDRALVFVDNHDNQRGHGAGGSSILTFWDARMYKMAVGFMLAHPYGFTRVMSSYRWNRNFQNGKDQNDWIGPPNNNGVTKEVTINADTTCGNDWVCE
Amy2b     LVPSDRALVFVDNHDNQRGHGAGGSSILTFWDARMYKMAVGFMLAHPYGFTRVMSSYRWNRNFQNGKDQNDWIGPPNNNGVTKEVTINADTTCGNDWVCE

                 410       420       430       440       450       460       470       480       490       500
Amy1 V1   HRWRQIRNMVAFRNVVNGQPFANWWDNDSNQVAFGRGNKGFIVFNNDDWALSETLQTGLPAGTYCDVISGDKVDGNCTGIKVYVGNDGKAHFSISNSAED
Amy1 V2   HRWRQIRNMVAFRNVVNGQPFANWWDNDSNQVAFGRGNKGFIVFNNDDWALSETLQTGLPAGTYCDVISGDKVDGNCTGIKVYVGNDGKAHFSISNSAED
Amy2a1
Amy2a2    HRWRQIRNMVAFRNVVNGQPFSNWWDNNSNQVAFSRGNRGFIVFNNDDWALSATLQTGLPAGTYCDVISGDKVDGNCTGLRVNVGSDGKAHFSISNSAED
Amy2a3    HRWRQIRNMVAFRNVVNGQPFSNWWDNNSNQVAFSRGNRGFIVFNNDDWALSATLQTGLPAGTYCDVISGDKVDGNCTGLRVNVGSDGKAHFSISNSAED
Amy2a4    HRWRQIRNMVAFRNVVNGQPFSNWWDNNSNQVAFSRGNRGFIVFNNDDWALSATLQTGLPAGTYCDVISGDKVDGNCTGLRVNVGSDGKAHFSISNSAED
Amy2a5    HRWRQIRNMVAFRNVVNGQPFSNWWDNNSNQVAFSRGNRGFIVFNNDDWALSATLQTGLPAGTYCDVISGDKVDGNCTGLRVNVGSDGKAHFSISNSAED
Amy2b     HRWRQIRNMVAFRNVVNGQPFANWWDNNSNQVAFSRGNRGFIVFNNDDWALSATLQTGLPAGTYCDVISGDKVDGNCTGLKVNVGSDGKAHFSISNSAED

                 510
Amy1 V1   PFIAIHAESKI
Amy1 V2   PFIAIHAESKI
Amy2a1
Amy2a2    PFIAIHADSKL
Amy2a3    PFIAIHADSKL
Amy2a4    PFIAIHADSKL
Amy2a5    PFIAIHADSKL
Amy2b     PFIAIHADSKL
```

Supplemental Figure 1. ClustalW alignments of predicted protein sequences of mouse amylase proteins: Amy1 V1 (NP_031472.2), Amy1 V2 (NP_001110505.1), Amy2a1 (XP_011238675.1), Amy2a2 (NP_001153624.1), Amy2a3 (NP_001153623.1), Amy2a4 (NP_001153622.1), Amy2a5 (NP_001036176.1) and Amy2b (NP_001177332.1). Bold indicates mis-aligned residues. 53 non-identical residues between Amy1 and the full-length Amy2 proteins (Amy2a2, Amy2a3, Amy2a4 and Amy2a5), including a 3 residue deletion in Amy2 proteins. All full-length Amy2 proteins are of identical sequence. Amy2b is of identical sequence except for deletion of 55 internal residues, plus a single residue substitution (A for S). Amy2a1 is a 3'-end truncated protein with an additional 35 residues at its 3'-end. ClustalW multiple alignment parameters: open gap penalty, 30; extend gap penalty, 5.0; delay divergent, 30%; gap distance, 4; similarity matrix, gonnet.
