# Supplementary material for: Murine Salivary Amylase Protects Against Streptococcus mutans-Induced Caries
Source: Front Physiol. 2021 Jul 2;12:699104. doi: 10.3389/fphys.2021.699104 (PMC8283412; doi:10.3389/fphys.2021.699104)
Supplement: Supplementary file 2 [file Table_1.DOCX]

**Supplemental Table 1.** Primers and PCR conditions in genotyping of *Amy1* KO mice. PCR reactions to produce Southern probe templates were run in Taq PCR Master Mix (Qiagen); 3 min at 98°C followed by 35 cycles (94°C, 30 s; 60°C, 30 s; 74°C, 30 s) and 3 min at 72°C. To verify correct insertion, PCR reactions with primer pairs P1 and P2 were run using the Epicentre Biotechnologies FailSafe PCR system with buffer E; 3 min at 94 °C followed by 30 cycles (94°C, 30 s; 55°C, 30 s; 72°C, 5 min) and 10 min at 72°C. To genotype mice with *neo* insert, reactions were run using the Epicentre Biotechnologies FailSafe PCR system with buffer B; 3 min at 94 °C then 30 cycles (94°C, 30 s; 55°C, 30 s; 72°C, 60 s) and 3 min at 72°C. To assess *neo* deletion and to genotype mice without *neo* insert, reactions were run using the Epicentre Biotechnologies FailSafe PCR system with buffer F; 3 min at 94 °C followed by 33 cycles (94°C, 33 s; 55°C, 30 s; 72°C, 180 s) and 5 min at 72°C. RT-PCR reactions for *Amy1* and *𝛽-actin* were run using the Epicentre Biotechnologies FailSafe PCR system with buffer E; 3 min at 94 °C followed by 35 cycles (*Amy1*) or 24 cycles (*𝛽-actin*) (94°C, 30 s; 52°C, 30 s; 72°C, 1.5 min) and 5 min at 72°C.

| **Primers** | **Primer Sequences** | **Product Size** |  |
| --- | --- | --- | --- |
| **Primers to produce Probes A and B** | | |  |
| Probe A | 5’-GTTGAAAGTAGAAGAGGTT-3’  5’-GTATCCTTTCACTATCCTCT-3’ | 229 bp |  |
| Probe B | 5’-TGTGAACAATTTCAAAAGGCA-3’  5’-CTCTGTGCTCTATAGAGGCTAA-3’ | 218 bp |  |
| **Primer Sets to Verify Correct Insertion and Sequence** | | | |
| P1F & P1R | For: 5’-GTCTCCTCTGGGCTACTAACTACAAATGGC-3’  Rev: 5’-ACCGCTATCAGGACATAGCGTTGGC-3’ | 4.20 kb |  |
| P2F & P2R | For: 5’-CCTGCGTGCAATCCATCTTGTTCAATGGC-3’  Rev: 5’-TTGGATATTGCACTCTGTGTGATGAGC-3’ | 3.39 kb |  |
| **Primers to Genotype Mice with *neo* Insert** | | |  |
| Forward - In *neo* | 5'-CGTCACCTTAATATGCGAAGTG-3' | KO: 638 bp  WT: 232 bp |  |
| Reverse – In 3’-Arm | 5'-CAGCATCTTGGTAGTTCTCGATAC-3' |  |  |
| Reverse – Intron 3 of WT | 5'-GACTCTAAGCTGCATTCAAGTACAC-3' |  |  |
| **Primers to Genotype Mice without *neo* after FLPe-Mediated Deletion** | | |  |
| Forward - In 5’-Arm | 5'-GTAACCTGGTCTGTTCATGTGACAAG-3' | KO: 681 bp  WT: 998 bp |  |
| Reverse - In FRT | 5'-TCGACGAAGTTCCTATTCCGAAG-3' |  |  |
| Reverse - 3’-end Exon 2 | 5'-CCTGCACACCTGCAAATCCATTAG-3' |  |  |
| **Primer Pair Targeting the R26^Fki^ allele [1]** | | |  |
| SD222 (R26^Fki^ allele) | 5'-CCCATTCCATGCGGGGTATCG-3' | 1.2 kb |  |
| SD223 (*ROSA26* locus) | 5'-GCATCTGGGAGATCACTGAG-3' |  |  |
| **RT-PCR Primers (*Amy1* and 𝛽-Actin)** | | |  |
| ***Amy1 V1*** | | |  |
| Forward - In Exon 1 | 5’-AGCTCAGATCACAGTGCTG-3’ | KO: 610 bp  WT: 1,075 bp |  |
| Reverse - Exons 6/7 | 5’-TCTCCCCAGTTCTTTAAGTAG-3 |  |  |
| ***Amy1 V2*** |  |  |  |
| Forward - In Exon 1 | 5’-CTCAACGTAAATCAGAAGATTC-3’ | KO: 518 bp  WT: 983 bp |  |
| Reverse - Exons 6/7 | 5’-CCAGTTCTTTAAGTAGGACATC-3 |  |  |
| ***Amy2a (2-5)*** | | |  |
| Forward - In Exon 3 | 5'-TGGGACTTTAACGATAAT-3' | 1,113 bp |  |
| Reverse - In Exon 11 | 5'-GCTTAATCCAAATCTCTT-3' |  |  |
| ***𝛽-Actin*** | | |  |
| Forward - In Exon 1 | 5'-GAGCACAGCTTCTTTGCAGCTC-3' | 223 bp |  |
| Reverse - In Exon 3 | 5'-CACCCACATAGGAGTCCTTCTGAC-3 |  |  |

[1[ F. W. Farley et al., 2000. Widespread recombinase expression using FLPeR (flipper) mice.

Genesis 28(3-4: 106-110.
